# Towards an understanding of somatosensory perturbation on covert speech production: a functional near infrared spectroscopy study

**DOI:** 10.3389/fnhum.2026.1757752

**Published:** 2026-05-07

**Authors:** Jacqueline Cummine, Dev H. Patel, Mitchell Holmes, Amberley Ostevik, Daniel Aalto

**Affiliations:** 1 Faculty of Rehabilitation Medicine, University of Alberta, Edmonton, AB, Canada; 2 Communication Sciences and Disorders, University of Alberta, Edmonton, AB, Canada; 3 Neuroscience and Mental Health Institute, University of Alberta, Edmonton, AB, Canada

**Keywords:** DIVA, functional near infrared spectroscopy (fNIRS), perturbation, somatosensory, supramarginal gyri (SMG)

## Abstract

**Introduction:**

Recent initiatives have sought to understand the impact of somatosensory feedback during varying speech production tasks. The supramarginal gyrus (SMG) is a multi-function region that represents the somatosensory system and plays an essential role in the DIVA model via the generation and monitoring of novel motor commands. Here, we investigate the extent to which SMG activity is modulated via somatosensory perturbations (i.e., oral anesthesia via lidocaine and oral stimulation via lollipop) during covert production tasks.

**Methods:**

Thirty adult participants completed two covert production tasks: a spelling judgment task that emphasized stored speech patterns (stored articulation; e.g., *bunt*) and a sound judgment task that emphasized constructing a new speech pattern (assembled articulation; e.g., *bont*). The tasks were performed under three perturbation conditions: lidocaine, lollipop and no perturbation. Brain activity was measured as oxygenated hemoglobin levels using functional near-infrared spectroscopy from the left hemisphere SMG, inferior frontal gyrus, dorsolateral prefrontal cortex, and inferior temporal gyrus, representing somatosensory, speech motor planning, task control, and visual input regions, respectively.

**Results:**

The lollipop perturbation resulted in significantly higher activity, compared to the lidocaine, in the left SMG (*p* = 0.001), inferior frontal gyrus (*p* = 0.01) and dorsolateral prefrontal cortex (*p* = 0.01). In addition, the lollipop perturbation showed higher functional connectivity between left SMG-inferior frontal gyrus when compared to the lidocaine condition but only for the stored production task.

**Conclusion:**

These findings provide evidence that the SMG is sensitive to alterations in oral sensory context even in the absence of overt articulation, supporting its proposed role within the DIVA model as a somatosensory state monitoring region that interacts with speech motor planning systems.

**Clinical trial registration:**

https://clinicaltrials.gov/study/NCT05854082?term=lidocaine%20speech&rank=1, Identifier, NCT05854082.

## Introduction

The Directions into Velocities and Articulators (DIVA) is a well-known model of speech production (Guenther and Vladusich, 2012; Guenther, 2006; Kearney and Guenther, 2019). One of the underlying assumptions of this model is that the generation of an articulator command is the summed result of feedforward (i.e., learned, highly familiar, internal, motor representations) and feedback commands (i.e., desired auditory and somatosensory targets; Kearney and Guenther, 2019). As such, changes to any of these command mechanisms have the potential to disrupt the generated articulatory output. In line with this notion, a growing body of literature points to notable changes in production behaviour following somatosensory disruptions (see Burke, 1975 and Cummine et al., 2021a for numbing-based studies using lidocaine; see Ito et al., 2009, 2016; Ito and Ostry, 2010; Ito and Ostry, 2012; and Namasivayam et al., 2022 for facial skin stretching; see Tremblay et al., 2003 for mechanical jaw perturbations; see Ito et al., 2024 for tongue perturbations), which have been useful in refining our understanding of the way speech production unfolds. However, the exploration of how such peripheral modifications to the somatosensory system impact the brain regions associated with speech remains relatively limited. The current study aims to address this gap by exploring the impact of somatosensory perturbations on brain activity and functional connectivity between regions associated with speech production.

### Perturbations to the speech system

There has been an increased presence of perturbation-based studies in speech literature where researchers are exploring how peripheral modifications (i.e., Grossman and Rajan, 2017; Halin, 2016; Lametti et al., 2012; Ogane et al., 2020; Van Den Bunt et al., 2017) impact behaviour (Ito et al., 2009; Ito and Ostry, 2010; Ito and Ostry, 2012). Such an approach provides a powerful means of probing the interaction between feedforward and feedback mechanisms. In a cross-modal repetition priming study, Namasivayam et al. (2022) showed that when participants’ mouths were shaped into articulatory positions corresponding to upcoming sounds (e.g., /d/ or /w/), their auditory speech recognition to words that began with /d/ (e.g., dance) or /w/ (e.g., wait), respectively, was facilitated. Notably, this effect was localized to low frequency spoken words (e.g., 1–10 per million), which are stimuli that reflect heightened engagement of feedback processes. These findings were argued to demonstrate that external somatosensory inputs influence word-level lexical processing, providing evidence for a connection between linguistic, speech perception and speech production based information. Similarly, Ogane et al. (2020) demonstrated that face stretching significantly influenced behaviour in an auditory lexical decision task, facilitating performance when there was congruence between the somatosensory face position and the auditory target, and decreasing performance when there was a mismatch between the somatosensory face position and the auditory target. Cummine et al. (2021a) examined how somatosensory perturbations influence covert speech by introducing oral disruptions to the mouth (i.e., a large lollipop or lidocaine). The authors reported condition-specific facilitation effects for the lollipop and lidocaine perturbations, particularly in tasks that depend heavily on feedback mechanisms, which was similar to Namasivayam et al. (2022). The authors suggested that oral somatosensory perturbations targeting the tongue selectively modulate speech processes that rely on feedback from sensory and motor regions. Together, this work demonstrates that altering somatosensory feedback offers a novel and promising avenue for elucidating the functional integration of feedforward and feedback pathways within speech networks.

### Somatosensory (supramarginal gyrus) and motor (inferior frontal gyrus) contributions to speech production

The impact of peripheral somatosensory perturbations on the central nervous system, albeit limited (Baciu et al., 2000 used a lip tube and MRI; Ito et al., 2016, 2021 used facial stretching and EEG; Golfinopoulos et al., 2011 used an inflatable balloon and MRI), has provided some insights into the interaction between brain regions that represent feedforward and feedback mechanisms. Particularly relevant to the current work is the study by Golfinopoulos et al. (2011), who implemented a clever somatosensory perturbation study, whereby participants’ jaws were unexpectedly adjusted via a balloon inflation while they were saying aloud visually presented nonwords in an MRI. This restriction in jaw closure served to induce a difference between the participant’s somatosensory expectation and the produced speech, resulting in a somatosensory error detection. Notably, the authors reported increased activity in the somatosensory regions, namely the bilateral supramarginal gyri (SMG), during the altered somatosensory experience (i.e., a restriction of the mouth closing due to balloon inflation), compared to trials where no somatosensory perturbation was provided. The SMG is a region long implicated in speech perception (Franken et al., 2022) and production processes (Kearney and Guenther, 2019; Meekings and Scott, 2021). According to the DIVA model (Tourville and Guenther, 2011), the target, error and state maps associated with the somatosensory feedback system are all represented in bilateral SMG. There is considerable evidence that these regions all activate when an individual is overtly or covertly producing speech (Mantegna et al., 2025). When a mismatch between expected and actual state maps occurs a signal is sent to the feedback control map, situated in the right ventral premotor cortex and right inferior frontal gyrus. In line with this notion, Golfinopoulos et al. (2011) examined shifts in connectivity (via structural equation modelling) between the bilateral anterior SMG and right ventral premotor cortex and right inferior frontal gyrus, during the perturbed speech compared to non-perturbed speech conditions. Notably, they reported an increase in connection strength between the right SMG and right IFG during the perturbed speech (path coefficient = 0.88) compared to non-perturbed speech (path coefficient = 0.78). These findings provide evidence for the activation and coordination of key speech regions during altered somatosensory feedback that is in line with the DIVA model.

Notably, Golfinopoulos et al. (2011) focused their analysis on the right SMG and right IFG, consistent with the DIVA model’s proposal that somatosensory feedback control mechanisms, including error computation and corrective signaling, engage parietal–frontal circuitry during overt perturbation (Tourville and Guenther, 2011). This approach is theoretically well-justified in overt production paradigms, where mechanical perturbations generate a true mismatch between expected and actual somatosensory state maps, thereby necessitating recruitment of the feedback control system to update motor commands. However, covert production paradigms present a fundamentally different computational scenario. In the absence of overt articulatory movement, no physical state map is instantiated and, therefore, no true peripheral mismatch occurs. Under strict interpretations of DIVA, this would predict minimal engagement of the somatosensory feedback control map in covert contexts.

At the same time, DIVA assigns target, state, and error representations within the somatosensory feedback system to bilateral SMG, suggesting that even in the absence of overt movement, internally simulated or predicted somatosensory states may still be activated (Tourville and Guenther, 2011). Covert speech production provides a powerful window into the feedforward and feedback mechanisms that underlie the speech network (Jackson et al., 2019; Sato et al., 2014; see also Perrone-Bertolotti et al., 2014 for a review on inner vs. overt speech). Neuroimaging and electrophysiological evidence demonstrate that covert and overt speech recruit largely overlapping cortical regions, which include the inferior frontal gyrus, superior temporal gyrus, premotor, and somatosensory cortices, indicating that internal speech planning engages the same predictive and monitoring systems that support overt articulation (Kell et al., 2017; Keller and Kell, 2016; Okada et al., 2018; Sato et al., 2014). For example, Novi et al. (2020) had participants overtly or covertly read a passage (using a blocked design) and found comparable activity in the left IFG (i.e., Broca’s) and left/right STG, albeit with an additional secondary later peak in the overt tasks ascribed to hearing one’s voice (Brumberg et al., 2016). Although overt production additionally activates regions associated with motor execution and auditory feedback, covert production isolates the internal generation and evaluation of speech motor commands without peripheral or acoustic confounds. Studies using various imaging approaches (i.e., fMRI, fNIRS, and electrocorticography) consistently show similar activation patterns during imagined and spoken language, with differences largely reflecting sensory consequences of articulation (e.g., hearing one’s own voice; Brumberg et al., 2016; Martin et al., 2016; Novi et al., 2020; Okada et al., 2018; Pei et al., 2011; Si et al., 2021).

In the context of DIVA, if covert production involves forward-model simulation of articulatory consequences then somatosensory target and state maps in the left SMG should still be engaged, and potentially modulated by peripheral somatosensory perturbations. This leads to several testable hypotheses. First, if covert tasks recruit internal forward models, somatosensory disruptions should alter SMG activity even without overt movement. Second, connectivity between the left SMG and frontal speech regions (e.g., IFG) should increase when somatosensory context is altered, reflecting adjustment of internally simulated state representations. Systematic examination of left hemisphere SMG activity and its functional connectivity during covert somatosensory perturbations therefore provides a critical opportunity to refine the DIVA model. Specifically, such work can determine whether feedback-related regions operate strictly as reactive controllers of overt movement or whether they also contribute to predictive simulation and state monitoring in the absence of motor execution. Demonstrating SMG engagement during covert disruption would extend DIVA by implicating somatosensory feedback mechanisms in internal forward modeling processes, whereas absence of such effects would support a more movement-contingent interpretation of the feedback control map.

### Input (inferior temporal gyrus) and task demands (dorsolateral prefrontal cortex)

Beyond the speech production network, other regions/networks support the fundamental processes associated with speech. Here we are particularly interested in the inferior temporal gyrus (ITG) and the dorsolateral prefrontal cortex (DLPFC). In the context of visually presented stimuli, the inferior temporal gyrus (ITG) serves as the input region to the network. Decades of work have shown that the ITG and surrounding regions are particularly sensitive to visual words, language, perception and the integration of visual data and multimodal sensory information (Cummine et al., 2016; Heim et al., 2009; Kuroki, 2006; Onitsuka et al., 2004a; Price, 2012). Although the premise of the DIVA model involves the activation of a speech sound map localized to the left premotor cortex (Kearney and Guenther, 2019) in response to an internally motivated utterance, a vast majority of speech production studies utilize externally driven inputs (i.e., the auditory and/or visual presentation of a word to be produced) in the experimental paradigm. In the context of an auditorily presented word, a framework associated with speech perception (i.e., see the ventral/dorsal model of auditory comprehension proposed by Hickok and Poeppel, 2007) must be considered to, at the very least, impact the input to the speech production system, which would be a shift from the premotor cortex (internally generated) to the auditory cortex (externally generated). Similarly, when a visual word is presented, a framework associated with reading (i.e., see the ventral/dorsal model of Cohen and Dehaene, 2009), needs to also be evaluated. Relevant to the current work, the extent to which a disruption to the speech network via the SMG, impacts other regions in the network, namely the ITG as an input region to the speech production system, remains to be seen.

The dorsolateral prefrontal cortex (DLPFC), on the other hand, is widely known to be involved in executive function and cognitive control, (Butler et al., 2023; Nejati et al., 2021), language acquisition and comprehension (Klaus and Schutter, 2018). What’s more, the DLPFC is involved in the integration of information from the visual, auditory and somatosensory cortex (Zhao and Ku, 2018); such findings are of particular importance for somatosensory perturbation studies that are aimed at changing the sensory and state map information from the feedback system, which may have consequences on regions responsible for integration. Here, we are interested in the extent to which a disruption to the somatosensory cortex (i.e., SMG) also impacts activity in the DLPFC.

### Functional connectivity between brain regions of the speech network

To fully appreciate the dynamic nature of the brain regions impacted by perturbations to the somatosensory speech system, it is necessary to consider the relationship (i.e., via functional connectivity) that exists between the disrupted region (i.e., SMG), and the other regions in the network (i.e., IFG, DLPFC, ITG; Golfinopoulos et al., 2011). The speech network is a highly complex and dynamic system that includes many nuances with respect to information cascading in a bottom-up and top-down manner as the generation, evaluation and decision making process unfold with respect to articulatory information (Cummine et al., 2016; Pei et al., 2011). And while perturbations may change activity in a ‘localist’ manner via increases or decreases in activity, task-based connectivity provides additional insight into the network. For example, the way in which the SMG communicates with the IFG in a context-dependent manner (i.e., with/without a somatosensory perturbation) is also likely impacted. In addition, we wanted to examine coupling between speech (SMG, IFG) and non-speech (DLPFC, ITG) regions during a task to better understand how these networks reconfigure in response to cognitive demand (i.e., stored articulation vs. assembled articulation). As mentioned previously, Golfinopoulos et al. (2011) reported shifts in connectivity between the SMG and feedforward regions (i.e., frontal-motor areas), with perturbed speech often showing an increase in connectivity strength compared to non-perturbed speech. Importantly, such subtleties in the speech network cannot be fully realized via behavioural changes, which may quickly adjust to task demands via compensatory strategies, but can be realized in brain imaging connections. Ultimately, an exploration of the task-based functional connections between these regions would provide additional insight into the speech network and the impact of somatosensory perturbations.

The presence of objects in the mouth to study somatosensory disruptions is not without its challenges with respect to safety (i.e., choking hazards) when utilizing brain imaging techniques that require a supine position (e.g., MRI). Functional near infrared spectroscopy (fNIRS) is an ideal approach to overcome such a barrier. FNIRS is an optical imaging technology that measures light absorption (between pairs of transmitter and receiver optodes) at the cortical level (Defenderfer et al., 2017; Pinti et al., 2015). More importantly, fNIRS provides a natural (i.e., namely sitting upright) and silent speech environment, with decent spatial (i.e., 2-3 cm) resolution (see Quaresima and Ferrari, 2019 for a review on fNIRS) of the speech network. While fNIRS is increasingly being used as a tool to explore overt/covert speech (Jackson et al., 2019; Moriai-Izawa et al., 2012; Novi et al., 2020; Si et al., 2021), the application of this methodological approach for exploring somatosensory perturbations is just being realized (Holmes et al., 2025).

### Purpose

Tongue-targeted somatosensory perturbations (i.e., lidocaine and lollipops) have been seen to affect decision-making tasks in covert speech production (Cummine et al., 2021a). However, the mechanism of action remains unknown. However, the effects of Golfinopoulos et al. (2011) raise several important questions. First, are shifts in the SMG activity (SMG-IFG connectivity), also found for covert speech? Considerable evidence has postulated that the speech network operates similarly in overt and covert paradigms (Mantegna et al., 2025); however, such claims need to be thoroughly tested. In this case, mismatches between expected and actual states would not be anticipated to engage the right lateralized feedback control map, but instead should be reflected in sensory and state maps of the left-hemisphere. Second, it is not known to what extent the somatosensory system differentiates between different types of perturbations (i.e., increased sensation via an object in the mouth vs. decreased sensation via a numbing agent)? In the auditory space, there is some evidence to suggest that the auditory feedback system continues to be active, even in the absence of feedback information (i.e., turning off a cochlear implant, Miller and Guenther, 2021). Whether such findings extend to the somatosensory system are not known. Finally, to what extent do tasks demands (i.e., increased demands on the feedforward vs. feedback systems) play a role in the activity/connectivity among regions associated with feedforward vs. feedback processes? Thus, to better understand the somatosensory feedback mechanism, neuroimaging of the speech network during somatosensory perturbations is needed. In this work, we use oral somatosensory perturbations (i.e., lidocaine and lollipop) to anesthetize and stimulate the oral cavity during covert speech production tasks. The study aims to explore the integration and involvement of the SMG, IFG, ITG and DLPFC, as a function of task demands (i.e., feedforward vs. feedback engagement) and peripheral perturbations (i.e., lidocaine vs. lollipops).

### Hypotheses

(1) In line with the DIVA model, and extending the work of Golfinopoulos et al. (2011) which focused on overt speech, we hypothesize that changes to the peripheral speech mechanism via somatosensory perturbations will be reflected in the somatosensory feedback region, namely the SMG, during covert speech tasks. (2) We anticipate that perturbations that increase (i.e., lollipop) vs. decrease (i.e., lidocaine) somatosensory information, will correspond to increases vs. decreases in SMG brain activity, respectively. (3) We expect tasks that increase the demands of the feedback system will show a greater impact of somatosensory perturbations. Specifically, in keeping with the DIVA premise that feedforward commands are summed with the sensory feedback-based commands to generate an overall motor command, we hypothesize that the node strength (i.e., sum of connection weights across all regions) will be higher for tasks that require greater feedback information and/or provide increased sensory-feedback information.

## Materials and methods

### Participants

The data reported here are part of a larger clinical trial research study that examined the effects of perturbations on behaviour and brain activity (see Holmes et al., 2025). Thirty participants of either sex (i.e., Female/Male) or identified gender (Woman/Man/Non-binary/Other) were recruited through advertisements posted on social media (e.g., Reddit) and from the University of Alberta mailing lists (e.g., Undergraduate student digest). All participants were adults (≥18 years; Mean age = 24, SD = 4.8; Females = 19), proficient in speaking English, and had no history of reading or language disorders. Exclusion criteria consisted of: participants with personal or family history of adverse reactions to anesthetics; individuals who cannot consume sugary products; severe kidney disease; severe liver disease; treatment with class I antiarrhythmic drugs (such as mexiletine) or class III antiarrhythmic drugs (such as amiodarone); lack of integrity of oral mucosa; allergy to non-medicinal ingredients and preservatives (and related compounds) of Lidocaine Viscous, such as methylparaben, propylparaben, paraaminobenzoic acid, saccharin, artificial colours and flavour; concomitant use of another anaesthetic containing lidocaine or another amide; participant being pregnant or suspecting that she might be pregnant. All participants were compensated with a $20 digital gift card for their participation. This clinical trial research study was approved and conducted in accordance with Health Canada (HC6-024-c234493) and the Research Ethics Board (REB) at the University of Alberta (Pro00088290).

### Materials

Assorted flavours of lollipops (Original Gourmet™; weight: ~31 g; circumference: ~11.2 cm) were used in the lollipop condition. The large circumference of these lollipops was necessary to prevent participants from moving the lollipop around the mouth during the covert production tasks. We used Lidocaine Viscous 2% (Lidocaine Hydrochloride Oral 220 Topical Solution USP) in the lidocaine condition.

### Tasks and stimuli

**Figure 1:**
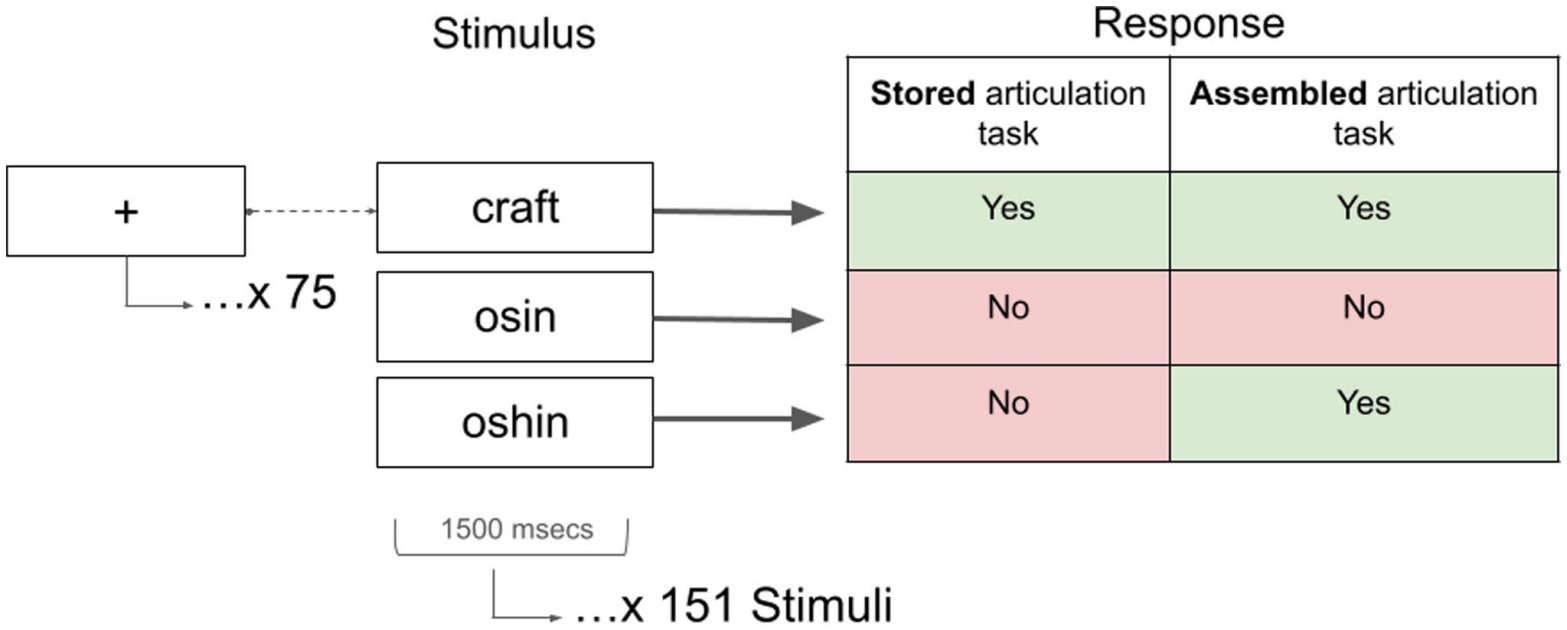
Schematic of the experimental paradigm. SA, stored articulation task; AA, assembled articulation task. Yes, green key; No, red key.

Two covert production tasks were employed with a lexical decision component to assess behaviour. We distinguish between stored articulation (SA), which relies on highly practiced, lexicalized articulatory plans, and assembled articulation (AA), which requires the online construction of an articulatory sequence, particularly for unfamiliar or novel stimuli. In the stored articulatory tasks (SA), participants were instructed to ‘press the green key if the letter string spells a real word’ and ‘press the red key if the letter string does not spell a real word’. In this task, participants were expected to respond with the green key (i.e., yes) to regular words (e.g., hint, ash) and irregular words (e.g., pint, ocean; i.e., stimuli that lack the explicit spelling-to-sound correspondence in English). The inclusion of these two types of stimuli did mean that participants could rely on highly familiar, stored, articulatory plans; however, participants had to generate an internal production to the stimuli, as a simple view of the spelling may prompt a red key (i.e., no) response to irregular words (i.e., ‘ocean’ pronounced using standard English spelling-to-sound correspondences may produce /ˈoʊtʃɛn/ or /ˈɑkɛən/). They were expected to respond with the red key to pseudohomophones (i.e., oshin) and nonwords (i.e., osin). In the assembled articulatory tasks (AA), participants were instructed to ‘press the green key if the letter string sounds like a real word’ and ‘press the red key if the letter string does not sound like a real word’. In this task, participants were expected to respond with the green key to regular words, irregular words *and* pseudohomophones. Again, the internal assembly of an articulation, particularly to the novel pseudohomophones, is necessary to ensure accurate green key (i.e., yes) responses to these stimuli (i.e., so ‘oshin’ is pronounced like /ˈoʊʃən/). While stored articulatory plans could be retrieved for the real words, the unfamiliarity of the pseudohomophones would require an internally assembled articulatory sequence (i.e., covert articulation), from which they could compare the auditory representations to make accurate decisions. Participants were expected to respond with the red key (i.e., no) to nonwords (see Figure 1). The English Lexicon Project (ELP) database was used to select 151letter strings for each task (SA and AA) with an equal number of real words and pseudowords (Balota et al., 2007). An event-related fNIRS design was employed, and as such, the 151 stimuli were intermixed with 75 rest trials (i.e., fixation) that were randomly presented throughout the experiment. Stimuli were presented using E-Prime v 3.0 (Psychology Software Tools, Inc, 2016), in black text on a white background, at the centre of a screen for 1,500 ms. The intertrial stimulus (i.e., the number of consecutive fixations presented) ranged from 0 s to 9,000 ms, and was variable throughout the experiment and across participants. Six unique versions of the tasks were created (i.e., two tasks (SA vs. AA) x 3 perturbations (lollipop, lidocaine, no perturbation)) to ensure there were no repeated stimuli among the tasks/perturbations (i.e., a total of 906 stimuli were presented across the two tasks and three perturbation conditions; see Cummine et al., 2021a for additional details on the task and stimuli characteristics).

### Functional near infrared spectroscopy (fNIRS)

**Figure 2:**
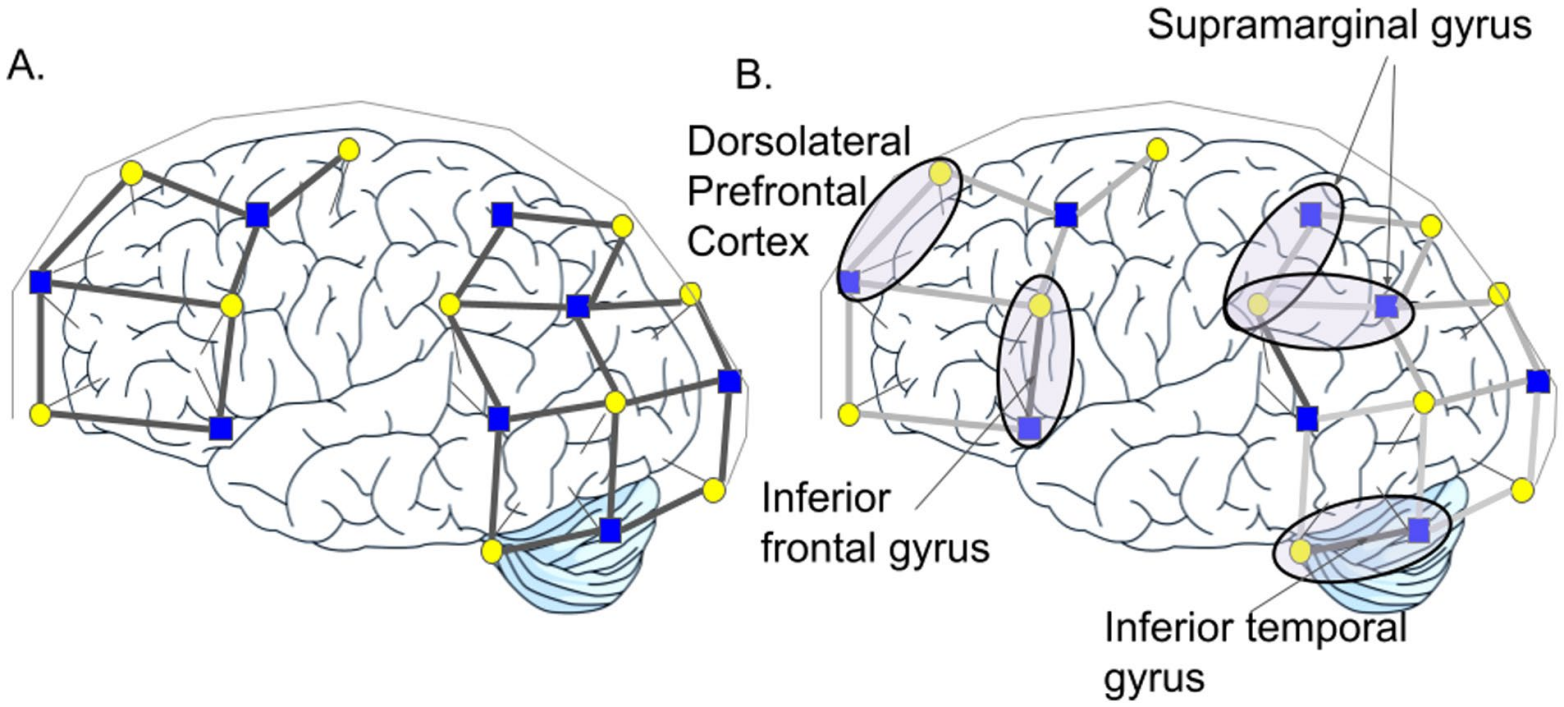
**(A)** Functional near infrared channel array on the left hemisphere. Blue square, receiver optode; yellow circle, transmitter optode. Each pair of optodes forms a channel from which oxygenated hemoglobin concentrations can be measured. **(B)** Regions of interest including the inferior frontal gyrus, supramarginal gyrus, dorsolateral prefrontal cortex, and inferior temporal gyrus.

Participants came to the lab where they were then measured for the fNIRS cap and its placement while seated in front of a computer. Placement of the cap was guided by the International 10–20 positioning system, beginning with two measurements of the head (i.e., tragus to tragus, inion to nasion) to find the mid-point of the skull (Cz). The fNIRS cap was aligned with this region, which then allowed for spatial specificity with respect to regions of interest (e.g., optodes aligned with the somatosensory cortex and/or with the frontal cortex). We used an Artinis Brite24 imaging device (Artinis Medical Systems, n.d.), in conjunction with the program Oxysoft, the software used to record the data measured with the fNIRS system. The Brite24 is a two-device system, with one device on either side of the head, enabling the concurrent acquisition of HbO concentrations in the brain. Each device has 10 transmitter optodes and 8 receiver optodes, resulting in a total of 45 optode pairs (i.e., channels), which were distributed across the frontal, temporal and parietal lobes of the right and left hemispheres. For the current study, only specific channels on the left hemisphere that corresponded to our regions of interest were used (see Figure 2). The International 10–20 positioning system ensured the positions of the sources and detectors could be scaled up or down to fit caps of different head sizes and record from the same cortical regions across participants. The fNIRS receivers were connected via bluetooth to a laptop where the signals were recorded and time locked to the presentation of stimuli. Once the optodes were in place, the researchers assessed the data acquisition (DAQ) values, which indicated the percentage of light being received by the receivers, to determine the initial signal quality of each channel. Due to time constraints, patient comfort, and battery power, researchers spent a maximum of 30 min to obtain the best signal quality possible, with the timer starting once the cap had been placed on the participant’s head.

### Procedure

Once the consent forms were read and signed, the participant was seated in front of a computer while measurements were taken for the fNIRS cap. The stimuli and instructions of the task were presented to the participants. For the duration of the task, participants in the lollipop condition were asked to hold the lollipop in the center of their mouths. Two breaks (i.e., after 75 stimuli) were provided for each task without any time restrictions to accommodate the accumulation of saliva and possible constraints on the jaw. After the break, participants continued the task by pressing the spacebar on the keyboard. In the lidocaine condition, the participants swished the Lidocaine Viscous 2% solution for 1 min, and the tasks began immediately after. To keep the experimental paradigm consistent, the same two breaks were provided in the lidocaine condition as was provided in the lollipop condition. Following the lidocaine condition, participants completed a survey with a scale of 1 to 10, where 10 represented maximum numbness to express the level of sensation that was lost due to lidocaine. Also, they were asked to indicate the areas of their mouths where they experienced this loss of sensation (such as the tongue, lips, cheeks, etc.). The no perturbation task was the control in the study where the participants completed each task without lollipop or lidocaine. The same two breaks were offered in the no-perturbation condition. In each condition, the order of the SA and AA tasks was randomized as was the lollipop and no-perturbation conditions. However, the lidocaine condition was applied last for all participants due to the prolonged anesthetic effects. To counteract potential order and fatigue effects, the study was split into two sessions for 9 participants (i.e., approximately 30%). One session included the lidocaine condition, and the other included the lollipop and the control conditions. The order of the sessions was randomized. The whole study was conducted in one session for the remaining 70% of the participants.

### fNIRS analysis

Data underwent signal quality analysis using the Quality Testing of Near Infrared Scans (QT-NIRS) toolbox. The QT-NIRS toolbox, developed by Hernandez and Pollonini (2020), calculates the scalp coupling index (SCI; threshold = 0.50; Si et al., 2021) and peak power (threshold = 0.1) for every five-second interval by computing the correlation between cardiac signals found in oxygenated and deoxygenated hemoglobin. For each task and condition, the QT-NIRS output consists of a bar graph of the channels that exceeded the threshold (i.e., channels that had good data quality). Further analysis was not conducted on any channel that did not reach the threshold.

fNIRS data was pre-processed using Homer3 version 1.33.0 (Matlab R2021b). First, the data was downsampled from 50 Hz to 10 Hz using the sampling rates documented in earlier research (Cui et al., 2012). Next, raw data intensity was transformed into optical density (OD) data (hmrR_Intensity2OD function; Yücel et al., 2016). Baseline shifts and high-frequency spikes were then detected at the channel level (hmrR_MotionArtifactByChannel) and motion was corrected using a Spline function (hmrR_MotionCorrectSplineSG) (Di Lorenzo et al., 2019; Jahani et al., 2018). A linear fit function (hmrR_PreprocessOD_LinearFit) was then applied, followed by a band-pass filter (hmrR_BandpassFilt) with a high-pass filter of 0.010 and low-pass filter of 0.50 to remove physiological noise. The corrected OD data were then converted to oxygenated (HbO_2_) and deoxygenated (HHb) hemoglobin concentration changes using the modified Beer–Lambert law (partial pathlength correction of 1.0; function: hmrR_OD2Conc). Lastly, task-evoked hemodynamic responses were estimated using Homer3’s hmrR_BlockAvg function, which extracts stimulus-locked epochs and computes the average HbO response across repeated trials within each condition. A time window of −2 to 20 s relative to stimulus onset was used, keeping in mind that the actual temporal resolution of fNIRS is still inherently limited by neurovascular coupling and the hemodynamic response (i.e., 4–6 s; Kleinschmidt et al., 1996). Using Microsoft Excel, the channels corresponding to the regions of interest (ROIs) of each participant were isolated, standardized/normalized (converted to z-scores) and a baseline correction applied by subtracting the mean signal prior to stimulus presentation (t < 0).

### Regions of interest

The SMG, DLPFC, ITG, and IFG were selected for analysis (see Figure 2B). ROIs were delineated based on the 10–20 cap array and optode placement. The SMG was composed of two channels with a subsequent spatial resolution of approximately 5–6 cm, while the DLPFC, ITG, and IFG were represented each by a single channel with a spatial resolution of approximately 3 cm (Klein, 2024). Averaging of the signals from the two SMG channels was completed after normalization and baseline correction.

To capture the neural response to the stimuli, the entire time window from −2 s to 20 s post stimulus onset was measured for each individual. In alignment with the temporal features associated with underlying neurovascular coupling mechanisms, namely that the hemodynamic response function (HRF) typically rises within 3 s of neural activation, peaks around 8 s post-stimulus onset and returns to baseline around 12 s after stimulus onset we chose to average the signal between 8 and 10 s following stimulus presentation for statistical analysis of brain activity. These features have been documented in imaging modalities such as fMRI (e.g., Friston et al., 1994) and fNIRS (e.g., Schroeter et al., 2006). The selected window for connectivity analysis was the timeseries between 4 and 12 s, which aligns with the canonical temporal dynamics of the hemodynamic response function. In cases where a participant’s data was missing or excluded as a result of the SCI analysis, the missing values were replaced using mean substitution. That is, the average oxygenated hemoglobin concentration of the corresponding task and condition across participants was used in its place. As an exception, if none of the conditions within a task met the SCI threshold at a given ROI, the data for that ROI-task combination was excluded entirely from the analysis, and no substitution was applied. After exclusions and substitutions were performed, the final sample size was N = 28 for the DLPFC, N = 29 for the SMG, N = 26 for the ITG, and N = 28 for the IFG.

### Statistical analysis

Statistical analysis of mean brain activity from the fNIRS data was completed using IBM SPSS Statistics (Version 29) predictive analytics software. For each participant, the normalized, baseline corrected brain signal time course (as a function of task (SA/AA), perturbation (lollipop/lidocaine/no perturbation) and region (inferior frontal gyrus/supramarginal gyrus, dorsolateral prefrontal cortex/inferior temporal gyrus)) was extracted and then averaged across participants. To test for differences in mean activity, a 2 (task: SA vs. AA) × 3 (perturbation: lidocaine, lollipop, none) × 4 (brain region: SMG, IFG, DLPFC, ITG) repeated measures ANOVA was run on the isolated and averaged the time series between 8 and 10 s post-stimulus onset (Logothetis et al., 2001). Significant effects are followed up with Bonferroni corrected paired samples *t*-tests. Finally, exploratory pairwise *t*-tests were run to test for effects of perturbations within each region and task. Both uncorrected and corrected via Benjamini-Hochberg, *p*-values are reported.

Functional connectivity was conducted via an ROI-to-ROI bivariate analysis (Nieto-Castanon, 2020). More specifically, for each participant, the time series between 4 to 12 s post-stimulus onset was correlated between each pair of ROIs. Correlations were then averaged across participants and tested against 0 to determine significance. In the interest of examining perturbation specific effects to the SMG, r-values were then transformed using a Fisher’s r to z-test to test whether the magnitude of the correlations differed between SMG-ROI pairs. Given the exploratory nature of this study and the constraints of the clinical trial (a pre-determined sample size of *N* = 30 participants), we recognize limits with respect to statistical power. As such, we report significant findings at a two-tailed *p* < 0.05 (uncorrected).

Node strength was calculated as the summation of all r-values (taking the absolute value of the negative correlations). To assess the relative importance of a connection during each task and perturbation, the r-values were transformed into z-values and the absolute values were calculated. A 3 (Perturbation: none, lidocaine, lollipop) × 2 (Task: stored vs. assembled articulation) × 6 (Connection: SMG-IFG, SMG-DLPFC, SMG-ITG, IFG-DLPFC, IFG-ITG, DLPFC-ITG) repeated measures ANOVA was conducted. Given the repeated nature of this analysis, only participants who had connections in all possible cells were included (N = 13). To minimize Type 1 error from this small sample, a Holm correction for family-wise error rate was applied.

## Results

**Figure 3:**
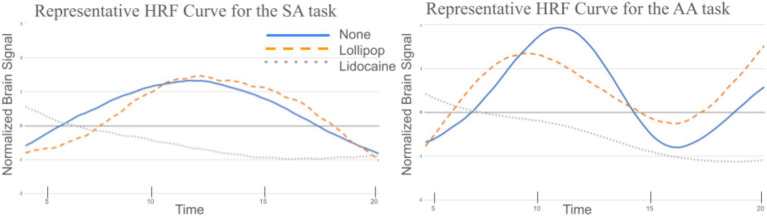
Representative hemodynamic response curves of fully processed data from the left supramarginal gyrus, as a function of condition and task, for one participant.

Due to an error in the collection software, responses to the AA of the control condition were not recorded for one participant at any ROI. Additionally, another participant chose not to complete the lollipop condition for either task. The mean brain signal for SA vs. AA trials, as a function of the perturbation, for a single participant, can be found in Figure 3 as a characterization of the unfolding of the brain signal over time.

### Behaviour

After the lidocaine condition, the participants were presented with the question: “On a scale of 1-10, how strong is the feeling of numbness (0 being no numbness and all feeling has returned and 10 being completely numb with no feeling).” A one-sample *t*-test against 0 found that participants reported a significant numbness (Mean = 4.47; SD = 1.76; *p* < 0.001).

The full behavioural analysis associated with this dataset can be found in Holmes et al. (2025). Relevant to the current study, a series of paired samples *t*-tests show that, in the SA task, participants responded faster compared to the AA task for each perturbation condition: (1) *No perturbation*–SA Mean = 706 ms (SD = 81) vs. AA Mean = 823 ms (SD = 65), (2) *Lollipop*–SA Mean = 716 ms (SD = 85) vs. AA Mean = 826 ms (SD = 79), and (3) *Lidocaine*–SA Mean = 704 ms (SD = 77) vs. AA Mean = 804 ms (SD = 84). In addition, participants responded more accurately in the SA task compared to the AA task for each perturbation condition, where the SA accuracy = 94% for all perturbation conditions. In contrast, the accuracy rates for the AA task were as follows: No perturbation = 83%, Lollipop = 84% and Lidocaine = 84% (all SA vs. AA pairwise tests on accuracy had *p*’s < 0.05). In addition, within the AA task, the Lidocaine condition produced significantly faster responses compared to the No perturbation condition, (*t*(26) = 2.14, *p* = 0.042, *d* = 0.41). No other statistically significant differences were found among the behavioural measures.

### Mean brain activity

Mean brain activity as a function of region, task and perturbation can be found in Table 1. The Mauchly’s test confirmed that the assumption of sphericity was met for all main effects and interactions (*p*’s > 0.05). There was a main effect of Perturbation, *F*(2, 44) = 8.35, *p* < 0.001, partial η^2^ = 0.275. Bonferroni corrected *t*-tests indicate that mean activity for the the lidocaine (M = −0.418; SE = 0.124) was significantly less than the lollipop (M = 0.298; SE = 0.141, *p* < 0.001) and but did not reach significance compared to no perturbation (M = −0.022, SE = 0.129, *p* = 0.052). There was a main effect of Region, *F*(3, 66) = 10.53, *p* < 0.001, partial η^2^ = 0.324. Bonferroni corrected *t*-tests indicate that compared to the ITG (Mean = −0.397; SE = 0.108, activity was greater in the SMG (Mean = 0.131; SE = 0.124, *p* = 0.003), IFG (Mean = −0.013; SE = 0.102, *p* = 0.020), and DLPFC (Mean = 0.089; SE = 0.086, *p* = 0.003). No other differences were found. There was no main effect of Task, *F*(1, 22) = 2.05, *p* = 0.167. None of the two-way nor three-way interactions were significant.

**Table 1:** Mean and standard deviations of brain activity as a function of task (SA, AA), brain region (SMG, IFG, DLPFC, ITG) and perturbation (lidocaine, lollipop, nothing).

Task	Region	Perturbation	Mean activity	Standard deviation
SA	SMG	Lidocaine	−0.3806	1.0075
		Lollipop	0.4685	1.0529
		Nothing	−0.0279	1.14512
	IFG	Lidocaine	−0.4751	0.92768
		Lollipop	0.3103	1.08398
		Nothing	−0.1372	1.05136
	DLPFC	Lidocaine	−0.4544	0.92977
		Lollipop	0.3301	1.19649
		Nothing	0.0437	0.91709
	ITG	Lidocaine	−0.5989	0.91833
		Lollipop	−0.3414	1.03388
		Nothing	−0.4632	1.03938
AA	SMG	Lidocaine	−0.1816	1.14529
		Lollipop	0.4602	1.25321
		Nothing	0.0656	1.37063
	IFG	Lidocaine	−0.2195	1.30261
		Lollipop	0.3692	1.04859
		Nothing	−0.2123	1.1854
	DLPFC	Lidocaine	0.1935	1.16454
		Lollipop	0.2436	1.12305
		Nothing	0.0434	1.28153
	ITG	Lidocaine	−0.9289	1.17119
		Lollipop	−0.3382	0.94502
		Nothing	0.0225	1.21393

**Figure 4:**
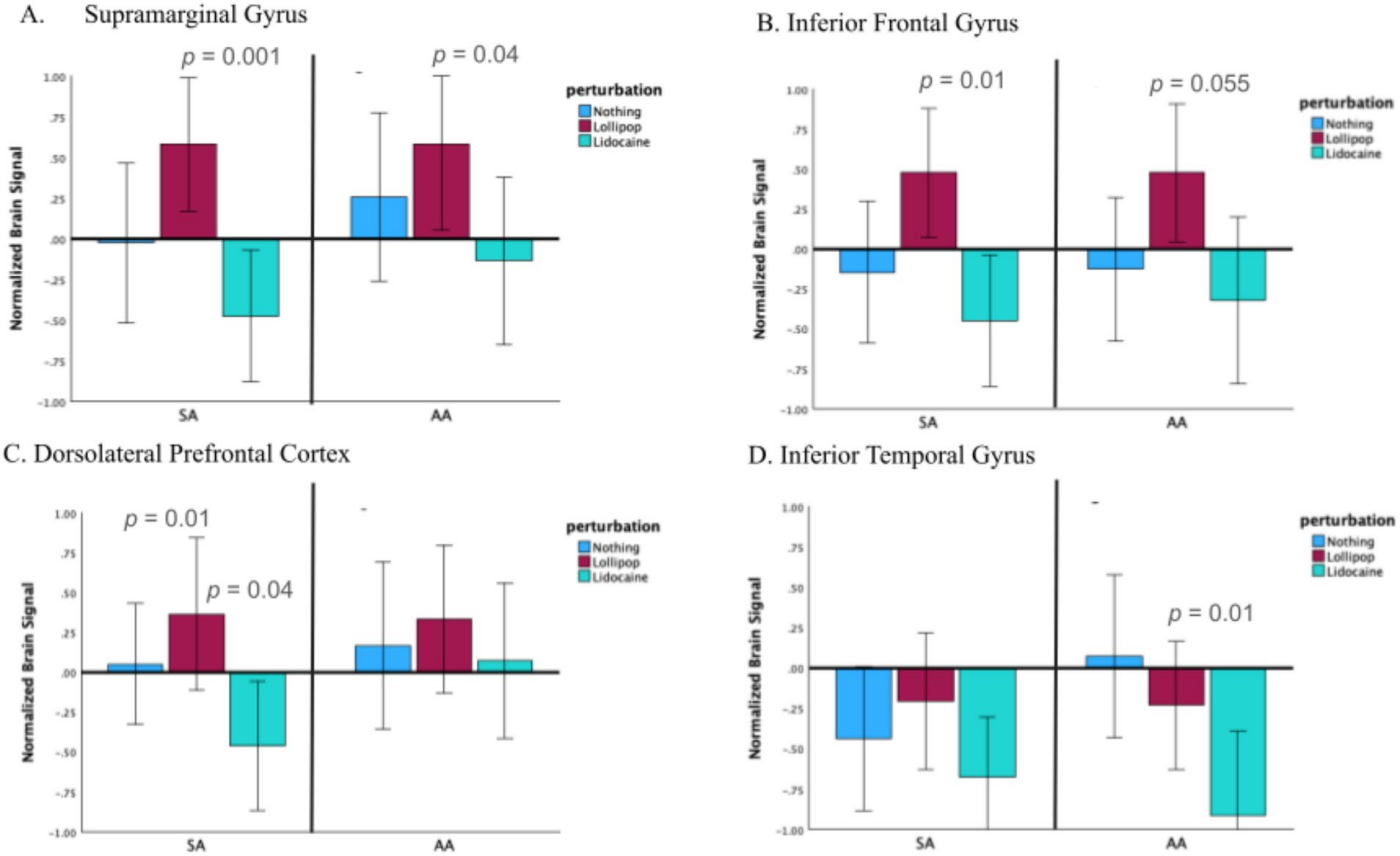
The mean percent brain signal as a function of task (SA vs. AA) and perturbation (lidocaine, lollipop, nothing) in the **(A)** supramarginal gyrus, **(B)** inferior frontal gyrus, **(C)** dorsolateral prefrontal cortex, and **(D)** inferior temporal gyrus. Error bars correspond to standard error. *p*-values correspond to two-tailed paired samples *t*-tests (uncorrected for multiple comparisons).

Exploratory paired samples *t*-tests were conducted to examine the extent to which perturbations impacted brain activity, in each task and as a function of region (Table 2). For the SA task, the lollipop had greater activity compared to the lidocaine in the SMG (0.469 vs. −0.381; Cohen’s d = 1.29, *p* = 0.001), IFG (0.310 vs. −0.475; Cohen’s d = 1.52, *p* = 0.01) and DLPFC (0.330 vs. −0.454; Cohen’s d = 1.53, *p* = 0.01). Each of these comparisons survived correction for multiple comparisons (see Table 2; see Figure 4). For the AA task, the lollipop had greater activity compared to the lidocaine in the SMG (0.460 vs. −0.182; Cohen’s d = 1.57, *p* = 0.037). In addition, the no perturbation condition had greater activity compared to the lidocaine condition in the ITG (0.023 vs. −0.925; Cohen’s d = 1.70, *p* = 0.010). These comparisons did not survive correction for multiple comparisons.

**Table 2:** Pairwise comparisons of the perturbation effect on brain activity as a function of task (SA, AA) and region (SMG, IFG, DLPFC, ITG).

Task	Region	Comparison	Original *p*-value	BH-corrected *p*-value
SA	SMG	Lidocaine-Lollipop	0.001+	0.012*
		Lidocaine-Nothing	0.161	0.276
		Lollipop-Nothing	0.095	0.228
	IFG	Lidocaine-Lollipop	0.011+	0.044*
		Lidocaine-Nothing	0.192	0.288
		Lollipop-Nothing	0.116	0.232
	DLPFC	Lidocaine-Lollipop	0.01+	0.044*
		Lidocaine-Nothing	0.04+	0.12
		Lollipop-Nothing	0.3	0.4
	ITG	Lidocaine-Lollipop	0.372	0.446
		Lidocaine-Nothing	0.57	0.57
		Lollipop-Nothing	0.559	0.57
AA	SMG	Lidocaine-Lollipop	0.037+	0.22
		Lidocaine-Nothing	0.459	0.687
		Lollipop-Nothing	0.287	0.526
	IFG	Lidocaine-Lollipop	0.055	0.22
		Lidocaine-Nothing	0.982	0.982
		Lollipop-Nothing	0.099	0.2376
	DLPFC	Lidocaine-Lollipop	0.858	0.936
		Lidocaine-Nothing	0.674	0.809
		Lollipop-Nothing	0.515	0.687
	ITG	Lidocaine-Lollipop	0.076	0.228
		Lidocaine-Nothing	0.01+	0.12
		Lollipop-Nothing	0.307	0.526

+significant at an uncorrected *p*-value of 0.05; *significant at a BH-corrected *p*-value for 12 comparisons.

### Functional connectivity of the SMG

**Figure 5:**
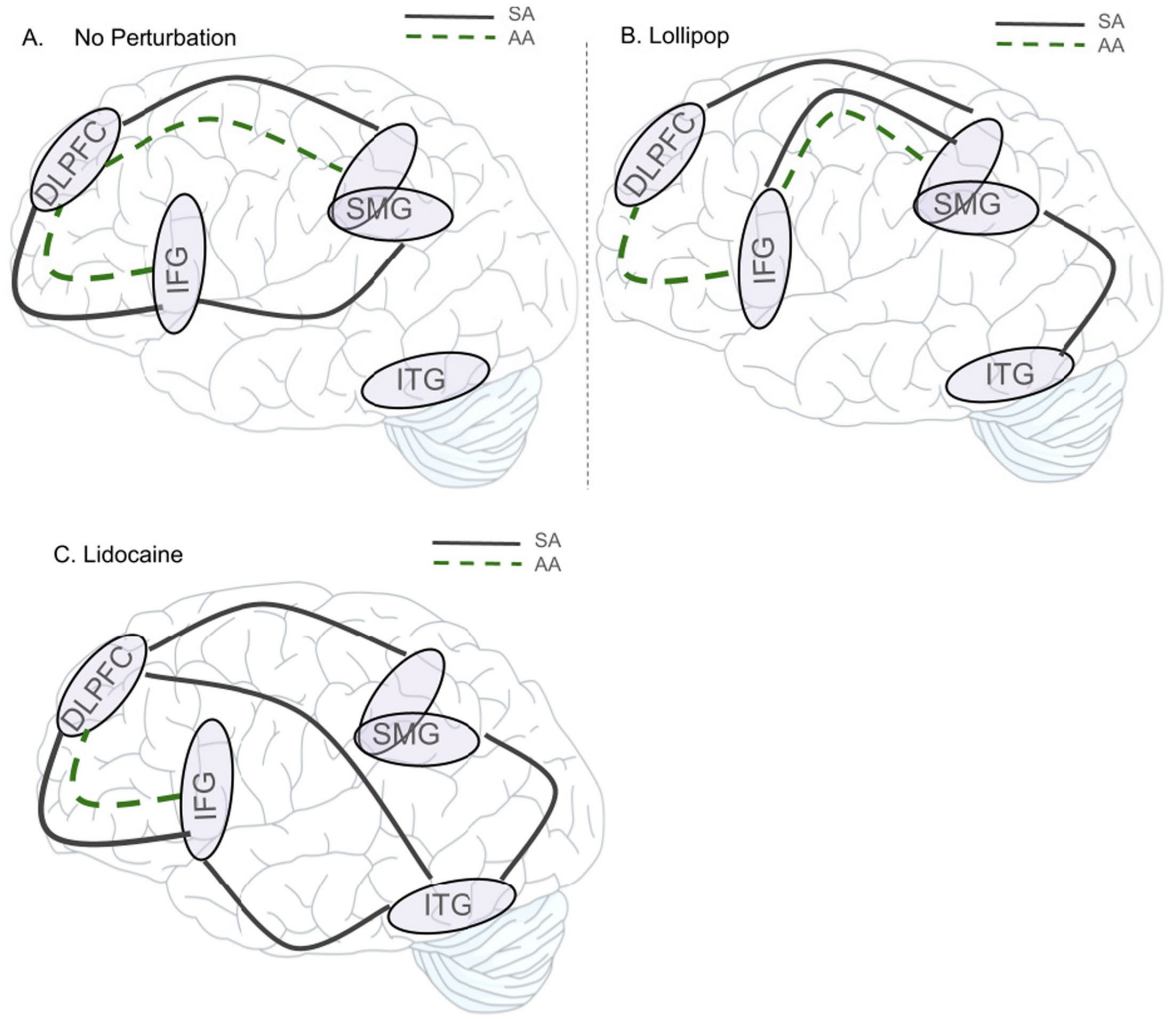
Functionally connectivity among the brain regions of interest (SMG, IFG, DLPFC, ITG) as a function of task (SA vs. AA) and perturbation condition: **(A)** No perturbation, **(B)** Lollipop, and **(C)** Lidocaine. Relationships are significant at *p* < 0.05(two-tailed).

**Figure 6:**
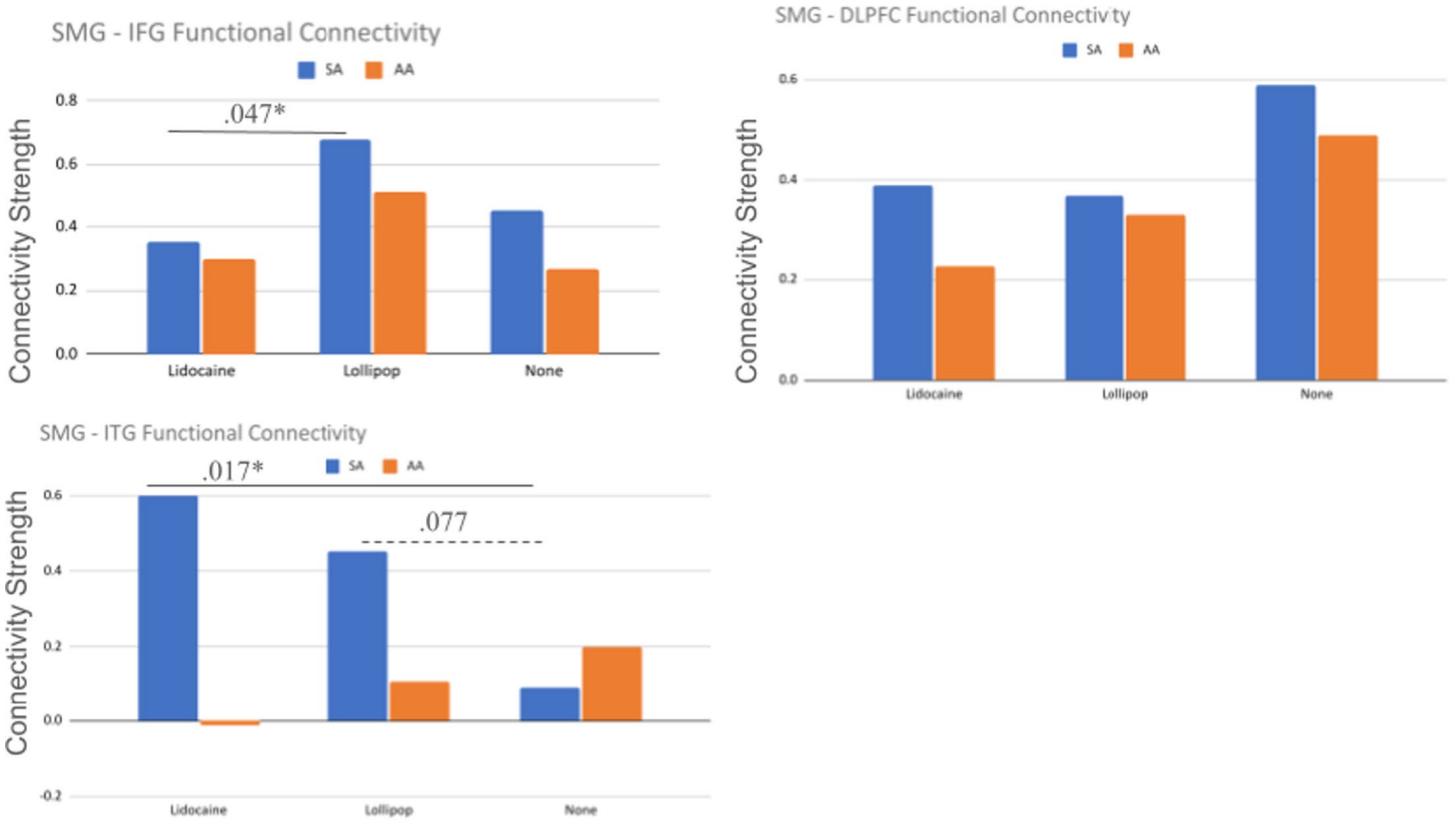
Comparison of functional connectivity between conditions. *Indicates a significant *z*-statistic (*p* < 0.05); SMG supramarginal gyrus; IFG inferior frontal gyrus; STG superior temporal gyrus; DLPFC dorsolateral prefrontal cortex; ITG inferior temporal gyrus.

The bivariate correlations as a function of region of interest, task and perturbation can be found in Table 3 (see also Figure 5). Overall, the DLPFC showed the highest number of significant relationships (10/18), followed by the SMG (9/18) and the IFG (9/18). These significant relationships spanned both the SA and AA tasks, and perturbation conditions (lidocaine, lollipop, no perturbation). The ITG had the fewest number of relationships (4/18), with zero relationships in the AA task. The magnitude of functional connectivity among SMG-ROI pairs was significantly higher for the lollipop vs. lidocaine conditions in the SMG-IFG, during the SA task (*p* = 0.047). The magnitude of functional connectivity among SMG-ITG pairs, during the SA task, was significantly higher for the lidocaine vs. no perturbation (*p* = 0.017), but did not reach significance for the lollipop vs. no perturbation (*p* = 0.077) (Figure 6). There were no statistically significant changes in functional connectivity strength associated with the AA task. There were also no significant changes in functional connectivity strength within the SMG-DLPFC pairs as a function of perturbation.

**Table 3:** Functional connectivity among regions.

			ROI					
			IFG		DLPFC		ITG	
		*Condition*	SA	AA	SA	AA	SA	AA
ROI	SMG	Lidocaine	0.351	0.299	0.389*	0.227	0.599*	−0.011
		Lollipop	0.679*	0.508*	0.365*	0.328	0.452*	0.108
		None	0.450*	0.266	0.588*	0.489*	0.093	0.198
	IFG	Lidocaine			0.804*	0.460*	0.875*	0.233
		Lollipop			0.189	0.486*	0.090	0.062
		None			0.785*	0.597*	0.230	0.033
	DLPFC	Lidocaine					0.788*	−0.076
		Lollipop					0.155	0.158
		None					0.288	0.184

*indicates correlation is significantly different from 0 (*p* < 0.05); SMG, supramarginal gyrus; IFG, inferior frontal gyrus; DLPFC, dorsolateral prefrontal cortex; ITG, inferior temporal gyrus.

### Functional connectivity across the regions

Node strength, calculated as the sum of all weights (i.e., r-values), was highest for the IFG (4.46) during the SA task, followed by the DLPFC (4.35), the SMG (3.97) and then the ITG (3.57). During the AA task, it was the DLPFC (3.05) with the highest node strength, followed by the IFG (2.95), SMG (2.41) and then the ITG (0.89).

A 3 (Perturbation: none, lidocaine, lollipop) x 2 (Task: stored vs. assembled articulation) x 6 (Connection: SMG-IFG, SMG-DLPFC, SMG-ITG, IFG-DLPFC, IFG-ITG, DLPFC-ITG) was conducted on the z-transformed node strengths. While there was no main effect of Perturbation (*p* = 0.710) or Task (*p* = 0.073), there was a significant main effect of Connection, *F*(5, 60) = 6.673, *p* < 0.001 (partial η^2^ = 0.05). Holm corrected follow-up tests confirmed that the DLPFC-IFG node strength (Mean = 2.24, SE = 0.168) was significantly higher than SMG-IFG (Mean = 1.824, SE = 0.168; Cohen’s d = 0.83, *p* = 0.045), DLPFC-ITG (Mean = 1.54, SE = 0.168; Cohen’s d = 1.40, *p* < 0.001) and ITG-IFG (Mean = 1.61, SE = 0.168; Cohen’s d = 1.26, *p* < 0.001). Of the two-way interactions, Task * Connection was significant, F(5, 60) = 3.341, *p* = 0.010 (partial η^2^ = 0.02), whereas Perturbation * Task (*p* = 0.983) and Perturbation * Connection (*p* = 0.732) were not significant. Holm corrected follow-up tests revealed that for the AA task, the DLPFC-IFG node strength (Mean = 2.43, SE = 0.2) was significantly greater than the SMG-ITG node strength (Mean = 1.34, SE = 0.2; *p* < 0.001). The same finding was not found for the SA task (DLPFC-IFG Mean = 2.05, SE = 0.2 vs. SMG-ITG Mean = 1.94; SE = 0.2). The three way interaction was not significant (*p* = 0.253).

## Discussion

Here we explored the neural activity and functional connectivity in various brain regions as a function of somatosensory perturbations during covert production. We utilized two task types (e.g., stored articulation and assembled articulation) and three perturbation methods (e.g., lidocaine, lollipop, and no perturbation). Several interesting findings emerged. First, we extend the work of Golfinopoulos et al. (2011) and show that the somatosensory feedback system of the DIVA model is modulated by peripheral perturbations during covert speech tasks. Second, in line with our hypotheses, the presence of a lollipop vs. lidocaine in the mouth corresponded to significant differences in mean brain activity in the SMG, IFG and DLPFC, with lollipop having greater activity compared to lidocaine in all comparisons. Third, with respect to functional connectivity, we saw partial support of our hypotheses. Specifically, the SMG-IFG connectivity was stronger in the lollipop vs. lidocaine perturbation but only in the SA task. Beyond our hypothesis, we noted that connectivity among the brain regions studied here was uniformly strong for the SA task, but more variable for the AA task (see Task * Connection node strength analysis). We discuss these findings in the context of the feedforward and feedback mechanisms of the DIVA model.

### Brain activity associated with somatosensory perturbations

The presence of a lollipop in the mouth corresponded to significantly higher activity in the SMG, compared to the lidocaine condition. While this difference did not reach statistical significance in relation to the no perturbation condition (likely a result of power), the general pattern of effects was for the lollipop to have higher mean activity overall, across both the SA and AA tasks. In addition, this same pattern was present in the left SMG and IFG, both speech targeted regions. This finding is in line with Golfinopoulos et al. (2011) perturbation study that saw an increase in SMG associated with an inflated balloon in the mouth, and supports the notion that the SMG is the locus of somatosensory information from the mouth. Importantly, we can also generalize the role of the SMG as a somatosensory region as there are several notable differences between the work of Golfinopoulos and the current study. First, we utilized a covert production task and examined the left SMG (feedback sensory, state and error map), whereas Golfinopoulos et al. (2011) employed an overt production task and studied the right SMG (feedback control map). In accordance with the DIVA model, we found that the left SMG was sensitive to somatosensory perturbations in covert tasks, which supports the claim that the left SMG is the location of the feedback sensory and state maps. These findings are in line with the hypothesis that feedback-related regions contribute to predictive simulation and state monitoring in the absence of motor execution. SMG engagement during covert disruption extends the DIVA by implicating somatosensory feedback mechanisms in internal forward modeling processes that are not movement-contingent. Second, Golfinopoulos et al. (2011) employed a task that delivered variable/unexpected perturbations, whereas we used a constant/expected perturbation approach. That both paradigms show increased activity in the left SMG is in line with the notion that the SMG is the locus of feedback state maps, which would respond to the sensory state alteration in comparison to baseline. The extent to which the left SMG is also sensitive to differences in prediction error (i.e., unexpected perturbations = large prediction error vs. expected perturbations = adaptation and reduced errors) would need to be explored in a future study that compared activity magnitude between unexpected and expected conditions. Third, we explored tasks that differentially rely on feedforward and feedback mechanisms. In contrast, Golfinopoulos et al. (2011) explored a single nonword production task that optimized feedback mechanisms. Interestingly, we were able to show that somatosensory perturbations impact the SMG in both types of tasks. Such findings point to an inherent automatic and tightly coupled state of the speech network. That is, brain activity within the speech network, namely the SMG, is impacted regardless of the potential necessity of their contribution to the task at hand. The presence of similar effects within the left SMG and left IFG (but not the DLPFC or ITG) further suggests that altered oral sensory context influences not only state encoding but also its integration with speech motor planning regions. This latter point also refutes claims that the effects reported here are due solely to altered arousal and/or attention because we see specific shifts in speech regions, but not non-speech regions. A generalized arousal/attention account would be more consistent with findings that showed an overall constant effect across tasks, perturbations, and regions. That these effects were observed even in covert task conditions, indicates that somatosensory feedback regions may participate in continuous state monitoring and predictive simulation rather than being recruited solely for overt error correction.

It is also worth noting that while the SMG and IFG demonstrated similar patterns of activity as a function of task and perturbation, this uniformity was not found in the ITG (the visual input region) and was differentially represented in the DLPFC during the AA task (i.e., see the lidocaine activity in Figure 4C). Given the similarity of mean activity effects within regions of the speech system (SMG and IFG; lollipop > nothing > lidocaine) vs. the variable effects outside the speech system (DLPFC and ITG), it appears that the perturbation manipulation reported here resulted in a speech-specific modulation rather than a general somatosensory response. Ultimately, additional work that explores additional speech-related and non-related regions, under varying conditions, is needed to fully support such claims.

A particularly novel contribution of the current study is the evidence provided for decreased activity in the SMG associated with the lidocaine perturbation. Similar to the lollipop, this decreased activity was found across both the SA and AA tasks and across regions (i.e., SMG, IFG, DLPFC). While previous work has explored the impact of numbing agents on the speech production system (Burke, 1975; Cummine et al., 2021); Niemi et al., 2006), there has been little brain imaging work in the area. Our initial claim for decreased activity during lidocaine, and increased activity during lollipop, was based on the noted role of the SMG within the DIVA model (Guenther and Vladusich, 2012; Kearney and Guenther, 2019). Specifically, the findings here are in line with the notion that the left SMG is the locus of the feedback sensory map. Indeed, we did find a significant main effect of perturbation that revealed mean activation was lowest under lidocaine, which was a manipulation designed to attenuate somatosensory input from the articulators, and highest under the lollipop condition, which enhanced tactile feedback. These differences in peripheral input dampen/amplify cortical processing demands, and possibly induce compensatory and/or heightened monitoring of articulatory feedback.

The extent to which somatosensory perturbations impact behavioural outcomes continues to be explored. For example, for tasks involving overt productions (Ashok Kumar et al., 2024; Burke, 1975; Lametti et al., 2012), there appears to be a negative consequence of somatosensory disruptions on speech performance (i.e., reduced accuracy). In contrast, speech perception work that has covert/button response has reported that somatosensory disruptions were associated with increased behavioural performance under conditions where the speech perception and face stretch were congruent (Ito et al., 2009, 2024). There is some evidence that individuals can compensate for the perturbation if the disruption is expected (Ashok Kumar et al., 2024; Ito et al., 2009, 2021, 2024; Lametti et al., 2012) vs. unexpected (Golfinopoulos et al., 2011; Ito et al., 2016). However, there is a necessary confound when trying to explore somatosensory perturbations in overt production studies as the most often utilized perturbation paradigms necessarily interfere with the articulatory system itself (i.e., face stretching, balloon inflation), making it difficult to disentangle the somatosensory feedback from the motor articulation. In our work, we have utilized covert production in an attempt to minimize this potential confound. Here we found that lidocaine resulted in faster response times (compared to no perturbation) but only in the AA task, replicating previous behavioural perturbation studies that show disruptions of somatosensory information primarily impact tasks (Cummine et al., 2021) and/or stimuli (Namasivayam et al., 2022) that rely on feedback mechanisms. Notably, the rated numbness associated with lidocaine was only 4.47/10, which may indicate that numbing had worn off quickly (i.e., these ratings were taken at the end of the lidocaine session) or that topical lidocaine does not fully numb the mouth. It remains to be seen whether full numbing procedures (i.e., injectable lidocaine) result in the same magnitude of behavioural effect seen here. And, while we are tentative in our conclusions about the behavioural consequences of our experimental paradigm, this finding is a replication of our previous work that found somatosensory perturbations induce facilitative effects under certain conditions (i.e., Cummine et al., 2021 reported 3 separate experiments). Further, these findings are consistent with studies that also utilize covert/button response paradigms (Ito et al., 2009, 2024). Given that the timing of the feedforward and feedback systems is difficult to examine with fNIRS, future research that employs EEG and/or MEG (i.e., see Mantegna et al., 2025) in conjunction with somatosensory perturbations would be particularly useful to map out the temporal envelope of feedforward and feedback systems (see Ito et al., 2016, 2021, 2024; Lankinen et al., 2024).

### Functional connectivity within the speech network

In keeping with the DIVA premise that feedforward commands are summed with the sensory feedback-based commands to generate an overall motor command, we explored the extent to which the node strength (i.e., sum of connection weights across all regions) was different for tasks that require greater feedback information (i.e., AA) and/or provide increased sensory-feedback information (i.e., lollipop). In line with the latter part of this hypothesis, we found that the SMG-IFG connection was stronger under the lollipop condition compared to the lidocaine condition, but only for the SA. We did not find support for the former part of our hypothesis, namely that node strength would be impacted by tasks that require greater feedback information (i.e., the AA task). In fact, we found no differences among the connections during the AA tasks, as a function of perturbation. Given the small number of participants that went into this exploratory analyses, these claims are highly preliminary and require further inquiry.

Although subtle, the perturbation effects found here were not uniform across the tasks when we considered connectivity (as opposed to mean activity reported above). With respect to the differences in magnitude of functional connectivity among SMG-ROI pairs, effects were only found for the SA task, but not the AA task. In addition, functional connectivity was comparatively high during the SA task vs. the AA task. More specifically, of the 18 pairwise (i.e., SA vs. AA) connectivity measures across all regions and perturbations, 15 of these correlations were of a higher magnitude in the SA condition vs. the corresponding AA condition. Somewhat in line with this, when we examined node strength, the only noted effect was found in the AA task, and it resembled a reduced SMG-ITG connection (compared to the DLPFC-IFG connection). Put another way, the SA condition had consistently strong connections across regions and perturbations. Together, these findings suggest that somatosensory perturbations can exert task-dependent effects on speech networks, influencing behavioural performance during feedback-weighted (AA) processing, while preferentially modulating network integration during feedforward-dominant (SA) processing. The fact that such nuances are not found in our localist mean activity analyses suggest that the relationships among the DIVA based regions is a more sensitive approach to capturing speech dynamics. Specifically, behavioural facilitation under lidocaine in the AA task may reflect altered error weighting and monitoring within already-engaged feedback control circuits possibly reducing irrelevant sensory input and/or altering attentional demands. On the other hand, perturbation-related changes in functional connectivity during the SA task indicate increased integration of somatosensory information into an otherwise stable feedforward architecture. The differences between connectivity magnitude and node strength may indicate that AA processing is characterized by selective reweighting of specific control pathways (e.g., DLPFC–IFG dominance over SMG–ITG), while SA processing engages more globally coordinated network adjustments. Ultimately, the specific subtleties of these various connections needs further examination via approaches that allow for the examination of interactions among regions (i.e., Structural equation modeling (SEM) as was done in Golfinopoulos et al., 2011) rather than co-activation as was done in the current study. Collectively, these patterns are consistent with the DIVA model’s proposal that feedforward and feedback subsystems operate in parallel but are differentially emphasized depending on task demands.

As mentioned in the introduction, the premise of the DIVA model involves the activation of a speech sound map localized to the left premotor cortex (Kearney and Guenther, 2019) in response to an internally motivated utterance, otherwise known as spontaneous speech. Yet, a vast majority of speech production studies utilize externally driven inputs (i.e., the auditory and/or visual presentation of a word to be produced) in the experimental paradigm. The extent to which spontaneous vs. externally motivated speech results in different activity and/or connectivity among speech regions needs further inquiry. In the current work, we included the ITG as the ‘input’ to the speech production network to examine the potential impact of a visually guided production. We did not see substantial recruitment and/or involvement of this region as a function of task or perturbation conditions. One of the main functions of the ITG is visual recognition, particularly involving objects, facial expressions, places, gestures, and complex shapes (Guidi et al., 2018; Price, 2012; Rolls, 2023). The ITG has been found to be involved in the recognition of orthographic letter strings–information that is passed on to various other brain regions depending on the nature of the task (Cummine et al., 2016). A potential explanation could be that the ITG’s primary function is the visual recognition of faces and objects. However, it can also aid in visual word and letter recognition within that function. Therefore, this region may aid the function of translating print into the speech network, but does not play a key role in subsequent processing. Interestingly, the presence of a perturbation (both lollipop and lidocaine) did produce an SMG-ITG connection, which was absent during the no perturbation condition, indicating that peripheral disruption to the speech network possibly invokes a compensatory mechanism and/or internal ‘check’ as the ‘typical’ connections are less reliable. Notably, the lidocaine condition also produced ITG-IFG and ITG-DLPFC connections, which suggests something inherently different about the reduced sensation that results in additional ITG connections to overcome the lack of somatosensory information. Finally, it is also noteworthy that all ITG connections were only found in the SA perturbation tasks, suggesting that connectivity changes resulting from peripheral manipulations may have a more substantial impact on automatized visual ‘input’ regions that supply information into sound and meaning-based brain regions (Taylor et al., 2019). To date, there is minimal information regarding whether there is a connection between the function of the ITG and the SMG as they pertain to translating print to speech. Future studies could attempt to distinguish whether the ITG has a direct or indirect role in the network.

### Future considerations

Throughout this study, we observed promising findings moving closer to understanding the inner neural mechanisms and functionalities of the impact of somatosensory perturbation in the speech network. However, several limitations of the current work mean these findings should be interpreted with caution and further explored in future studies. For example, we did not utilize any mechanism to ‘objectively’ verify covert speech engagement. Future work that combines electromyography (EMG; i.e., Nalborczyk et al., 2020) with fNIRS would be one way to more confidently make claims about brain activity associated with covert speech. In addition, while focusing on one region at a time can be beneficial in discovering what causes the change in function and activity moving from one task to another, this approach does not capture network dynamics. Future work that applies graph theory or dynamic causal modelling would be needed to elucidate the effects of somatosensory perturbations at the network level. In line with the notion of broader network analysis, utilizing a larger array of regions would allow for further understanding of the DIVA model. More specifically, the feedback control maps of the DIVA model are right lateralized (e.g., right ventral premotor cortex; Tourville and Guenther, 2011) and an examination of how these regions adjust to various somatosensory perturbations, whether expected/sustained, as in the current study, or unexpected/random, as was done in Golfinopoulos et al. (2011), would also be beneficial. These areas of inquiry would provide additional specificity regarding the nature of the connections reported here, and would serve to clarify the importance of ‘connection strength’ among the speech regions as they relate to behavioural outcomes. Finally, the nature of the clinical trial prohibited us from collecting more than 30 participants. Future work would benefit from an increased sample size, which would provide data that can be generalizable, leading to more concrete and clear conclusions.

## Conclusion

In sum, the present findings provide novel evidence that somatosensory perturbations modulate core speech-network regions during covert production and do so in a task-dependent manner consistent with the architecture of the DIVA model. Enhanced tactile input (lollipop) increased activity within left SMG and IFG, supporting the role of the SMG as a locus of somatosensory state representation and suggesting that feedback-related regions contribute to predictive state monitoring even in the absence of overt movement. At the network level, perturbation selectively strengthened SMG–IFG connectivity during stored articulation (SA), while assembled articulation (AA) was characterized by greater variability and selective pathway reweighting rather than global increases in coupling. Behavioural facilitation under lidocaine in the AA task further indicates that somatosensory disruptions influence feedback-weighted processing differently than feedforward-dominant processing. Together, these results support the DIVA model’s proposal that feedforward and feedback subsystems operate in parallel but are differentially emphasized depending on task demands, and extend the model by suggesting that somatosensory feedback mechanisms may function as continuous state monitors rather than solely reactive error-correction systems. Future work using temporally sensitive methodologies and broader network analyses will be critical to further refining our understanding of how altered peripheral sensory states shape internal forward models and the integration of sensory and motor representations during speech.

## Data Availability

The raw data supporting the conclusions of this article will be made available by the authors, without undue reservation.
